# De novo transcriptome sequence and identification of major bast-related genes involved in cellulose biosynthesis in jute (*Corchorus capsularis* L.)

**DOI:** 10.1186/s12864-015-2256-z

**Published:** 2015-12-15

**Authors:** Liwu Zhang, Ray Ming, Jisen Zhang, Aifen Tao, Pingping Fang, Jianmin Qi

**Affiliations:** Key Laboratory for Genetics, Breeding and Multiple Utilization of Crops, Ministry of Education / College of Crop Science, Fujian Agriculture and Forestry University, Fuzhou, 350002 China; Department of Plant Biology, University of Illlinois at Urbana-Champaign, Urbana, IL 61801 USA; Center for Genomics and Biotechnology, Haixia Institute of Science and Technology, Fujian Agriculture and Forestry University, Fuzhou, 350002 China

**Keywords:** Jute (*Corchorus* spp.), Transcriptome sequence, Cellulose synthesis, SNP

## Abstract

**Background:**

Jute fiber, extracted from stem bast, is called golden fiber. It is essential for fiber improvement to discover the genes associated with jute development at the vegetative growth stage. However, only 858 EST sequences of jute were deposited in the GenBank database. Obviously, the public available data is far from sufficient to understand the molecular mechanism of the fiber biosynthesis. It is imperative to conduct transcriptomic sequence for jute, which can be used for the discovery of a number of new genes, especially genes involved in cellulose biosynthesis.

**Results:**

A total of 79,754,600 clean reads (7.98 Gb) were generated using Illumina paired-end sequencing. De novo assembly yielded 48,914 unigenes with an average length of 903 bp. By sequence similarity searching for known proteins, 27,962 (57.16 %) unigenes were annotated for their function. Out of these annotated unigenes, 21,856 and 11,190 unigenes were assigned to gene ontology (GO) and euKaryotic Ortholog Groups (KOG), respectively. Searching against the Kyoto Encyclopedia of Genes and Genomes Pathway database (KEGG) indicated that 14,216 unigenes were mapped to 268 KEGG pathways. Moreover, 5 *Susy*, 3 *UGPase*, 9 *CesA*, 18 *CSL*, 2 *Kor* (*Korrigan*), and 12 *Cobra* unigenes involving in cellulose biosynthesis were identified. Among these unigenes, the unigenes of comp11264_c0 (*SuSy*), comp24568_c0 (*UGPase*), comp11363_c0 (*CesA*), comp11363_c1 (*CesA*), comp24217_c0 (*CesA*), and comp23531_c0 (*CesA*), displayed relatively high expression level in stem bast using FPKM and RT-qPCR, indicating that they may have potential value of dissecting mechanism on cellulose biosynthesis in jute. In addition, a total of 12,518 putative gene-associate SNPs were called from these assembled uingenes.

**Conclusion:**

We characterized the transcriptome of jute, discovered a broad survey of unigenes associated with vegetative growth and development, developed large-scale SNPs, and analyzed the expression patterns of genes involved in cellulose biosynthesis for bast fiber. All these provides a valuable genomics resource, which will accelerate the understanding of the mechanism of fiber development in jute.

**Electronic supplementary material:**

The online version of this article (doi:10.1186/s12864-015-2256-z) contains supplementary material, which is available to authorized users.

## Background

Jute fiber is second to cotton in its importance of natural fiber production in the world, which belongs to a member of the *Corchorus* genus in the Tiliaceae family. Unlike cotton, jute fiber is a bast fiber and isolated from the stem. There are almost 60 species in the genus *Corchorus*. Among these, white jute (*C. capsularis*) and dark jute (*C. olitorius*) are cultivated as crops and both are diploid species (2n = 14). Xiong et al. [1] suggested that the origin center of white jute is said to be Indo-Burma while the one of dark jute is Africa using morphological traits. However, Kundu et al. [2] recently reported that the two cultivated species of jute originated in Africa using nuclear and chloroplast simple sequence repeats (SSRs) or microsatellites. Though the origin of them remain contended, the two cultivated species are different in terms of growth habitat, disease resistance, and characteristics associated with flowering and silique shape. As one of the most important crops for natural fiber production, jute is mainly distributed in Bangladesh, India, China and east-central Africa. Since jute fiber is a biodegradable, renewable and environment-friendly cellulose fiber, it called golden fiber [1–3]. Therefore, jute has received much attention in many countries.

Sequencing and analysis of expressed sequence tags (ESTs) have been widely used for gene expression profiling, molecular marker discovery, and so on. To obtain the EST information, a bunch of transcriptome sequences based on the next generation sequencing (NGS) have been conducted for *Populus tomentosa* [4], *Ricinus communis* [5], *Vitis vinifera* [6] and some natural fiber crops [7–10]. Liu et al. [7] discovered 43,990 unigenes and 51 CesA genes using transcriptomic sequencing in ramie (*Boehmeria nivea L. Gaud*). Mudalkar et al. [9] used NGS to generate 53,854 contigs and identified the genes involved in lipid metabolism in flax. However, compared with these gelatinous-type natural fibers of flax (*Linum usitatissimum*), hemp (*Camelina sativa*) and ramie, jute fiber is a xylan-type fiber, similar to kenaf (*Hibiscus cannabinus*) (Mikshina et al. 2013). It is hard to conduct the genetic studies on fiber development in jute using these transcriptomic sequences of gelatinous-type natural fibers. To date, the studies on the transcriptomes of jute using NGS are still limited [11, 12]. Chakraborty et al. [11] conducted the bast transcriptomes of a *deficient lignified phloem fibre* mutant and its wild-type jute (*Corchorus capsularis*) using Illumina paired-end sequencing and obtained a total of 34,163 wild-type and 29,463 mutant unigenes in jute. Biswas et al. [12] identified a number of defense genes involved in the defense-response to *Macrophomina phaseolina.* using NGS in jute. For jute without a reference genome sequence, it is still not sufficient for functional genomics studies although there is one research of de novo assembly using NGS in jute.

In addition, the development of SNP (single nucleotide polymorphism) markers for jute has also lagged behind that in major crops. Few genetic studies on SNP markers development were reported to date. Biswas et al. [13] constructed a linkage map comprising 48 simple sequence repeats (SSRs) and 410 (SNPs) covering 2016 cM with a mean density of 4.2 cM per locus in a RIL population in white jute (*C. capsularis*). Thus, the scarcity of SNP markers and the resulting limited data for specific genes for fiber-related traits have resulted in a large knowledge gap for the improvement of jute fiber yield and quality.

There are three major objectives for the present study: (1) to characterize the transcriptome of jute and generate a broad survey of genes associated with vegetative growth and development; (2) to develop a large-scale data set of SNPs from transcriptomic sequences; (3) to identify the expression profiles of bast-related genes involved in cellulose biosynthesis. The discovered genes and newly developed SNPs will not only facilitate genomic studies, but also enrich molecular markers available for jute. The identified bast-related genes will provide a valuable knowledge to study cellulose biosynthesis.

## Result

### De novo assembly of transcriptome

**Fig. 1:**
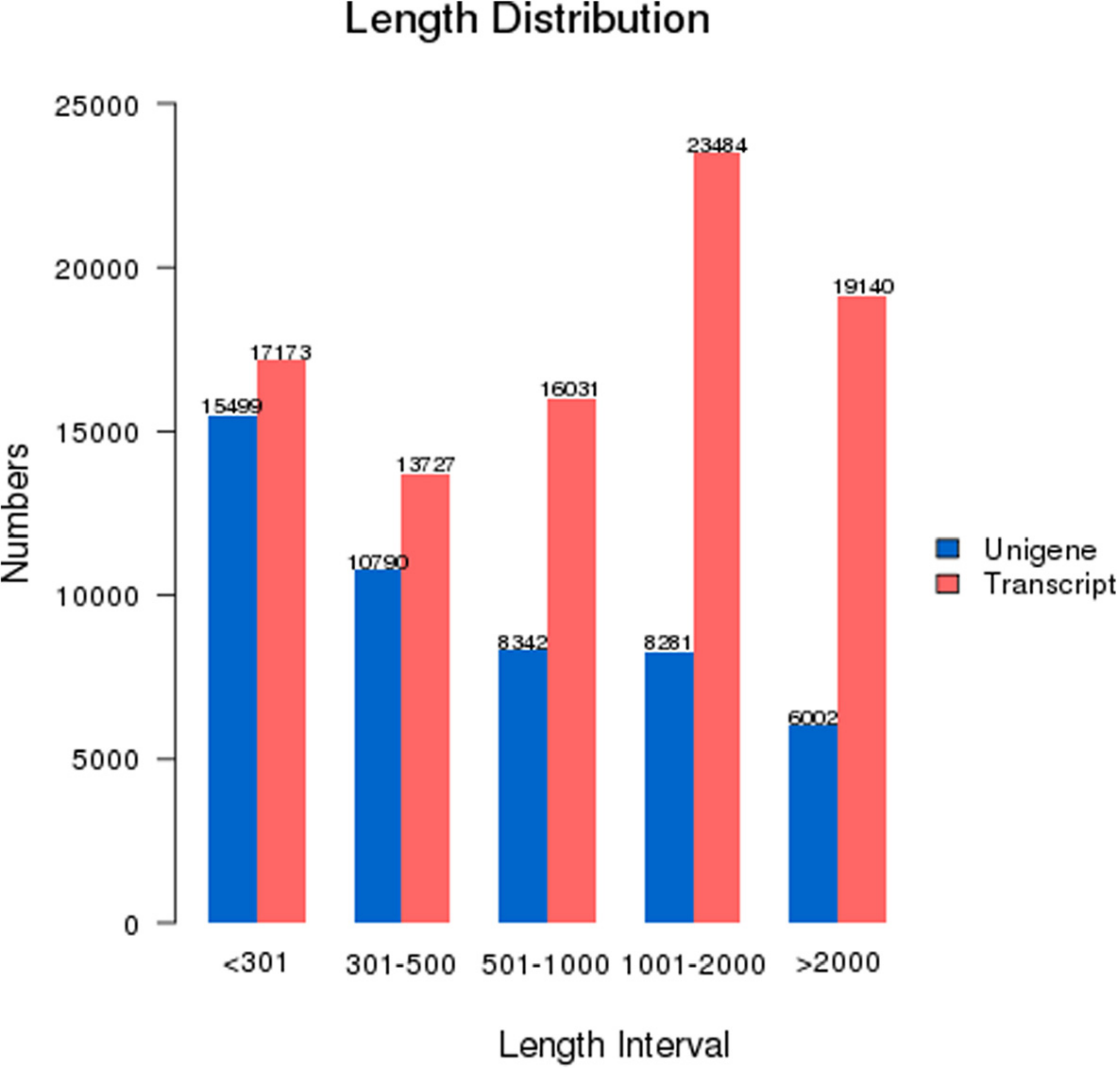
Length distribution of assembled transcripts and unigenes in jute. The horizontal axis is the length of transcripts and unigenes; and the vertical axis is the number of transcripts and unigenes

To characterize the transcriptome of jute and obtain a number of genes associated with vegetative growth and development, total RNA was pooled from diverse tissues of leaves, roots, stem bast and stem stick at a vigorous vegetative growth stage. Using Illumina paired-end sequencing, 79,754,600 clean reads with 98.27 % Q20 bases and 7.98 Gb with 43.48 % GC ratio were obtained, including 8,260,313 clean reads from stem bast (Table 1). The Trinity assembler [14] was used for *de novo* assembly of the clean reads. A total of 89,555 transcripts were assembled with an average and N50 length of 1290 and 2060 bp respectively (Fig. 1, Additional file 1). Of these, 52.4 % transcripts had lengths ranged from 200 to 1000 bp.

**Table 1:** Summary of data generated in the transcriptome sequence of jute

Samples	Raw reads	Clean reads	Clean bases	Error(%)	Q20(%)	Q30(%)	GC(%)
left.fq	41,242,545	39,877,300	3.99G	0.02	98.68	95.6	43.45
right.fq	41,242,545	39,877,300	3.99G	0.03	97.85	93.87	43.51
CK_JSB	8,269,487	8,260,313	0.62G	0.03	94.62	87.84	43.13

For one paired-end(PE) cDNA library (left.fq and right.fq), which was generated from pooled equal quantities of total RNA from stem bast, stem stick, leaves, and roots, the assembled sequence data have been deposited at the NCBI Sequence Read Archive (SRA, http://www.ncbi.nlm.nih.gov/Traces/sra) under the accession number SRP060467 vide BioSamples SRS980707. For a sample sequencing data from stem bast (CK_JSB), the SRA files bearing with the accession number SRP060467 vide BioSamples SRS1047357 are deposited in NCBI

Transcripts were assembled into unigenes, yielding 48,914 unigenes with an average length of 903 bp and the N50 length of 1703 bp respectively (Fig. 1, Additional file 2). The length of these unigenes varied from 201 to 15,535 bp. There were 26,289 unigenes (53.75 %) in the length range of 201 to 500 bp, 8342 unigenes (17.05 %) in the length range of 501 to 1000 bp, and 14,283 unigenes (29.20 %) with length more than 1000 bp (Fig. 1).

As mentioned above, there is no reference genome sequence for jute so far. To evaluate the quality of these assembled unigenes, the de novo assembled transcriptome sequence by Trinity was regarded as a reference sequence. All the clean reads were mapped to the assembled unigenes using the software of RSEM [15]. The 70,376,140 clean reads (88.24 %) were successfully realigned to the reference sequence, demonstrating that the quality of these assembled unigenes is sufficient to conduct the following analyses.

### Functional annotation

To identify the functions of these unigenes, sequence similarity search was conducted in the non-redundant protein sequences available at various databases using BLASTx (Basic Local Alignment Search Tool) with an E-value threshold of 10^−5^ [16] (Table 2). Of 48,914 unigenes, 25,739 (52.62 %) and 19,741 (40.35 %) unigenes displayed significant similarity to known proteins in Nr and SwissProt databases, respectively. Together, 27,962 (57.16 %) unigenes were annotated in at least one public databases such as Nr, KO, SwissProt, PFAM, GO, or KOG.

**Table 2:** The number and frequencies of unigenes annotated in the public databases

Search item	Number of unigenes	Percentage (%)
Annotated in nr	25739	52.62
Annotated in nt	13864	28.34
Annotated in KO	8460	17.29
Annotated in SwissProt	19741	40.35
Annotated in PFAM	19855	40.59
Annotated in GO	21856	44.68
Annotated in KOG	11190	22.87
Annotated in all databases	4183	8.55
Annotated in at least one database	27962	57.16
Total Unigenes	48914	100

**Fig. 2:**
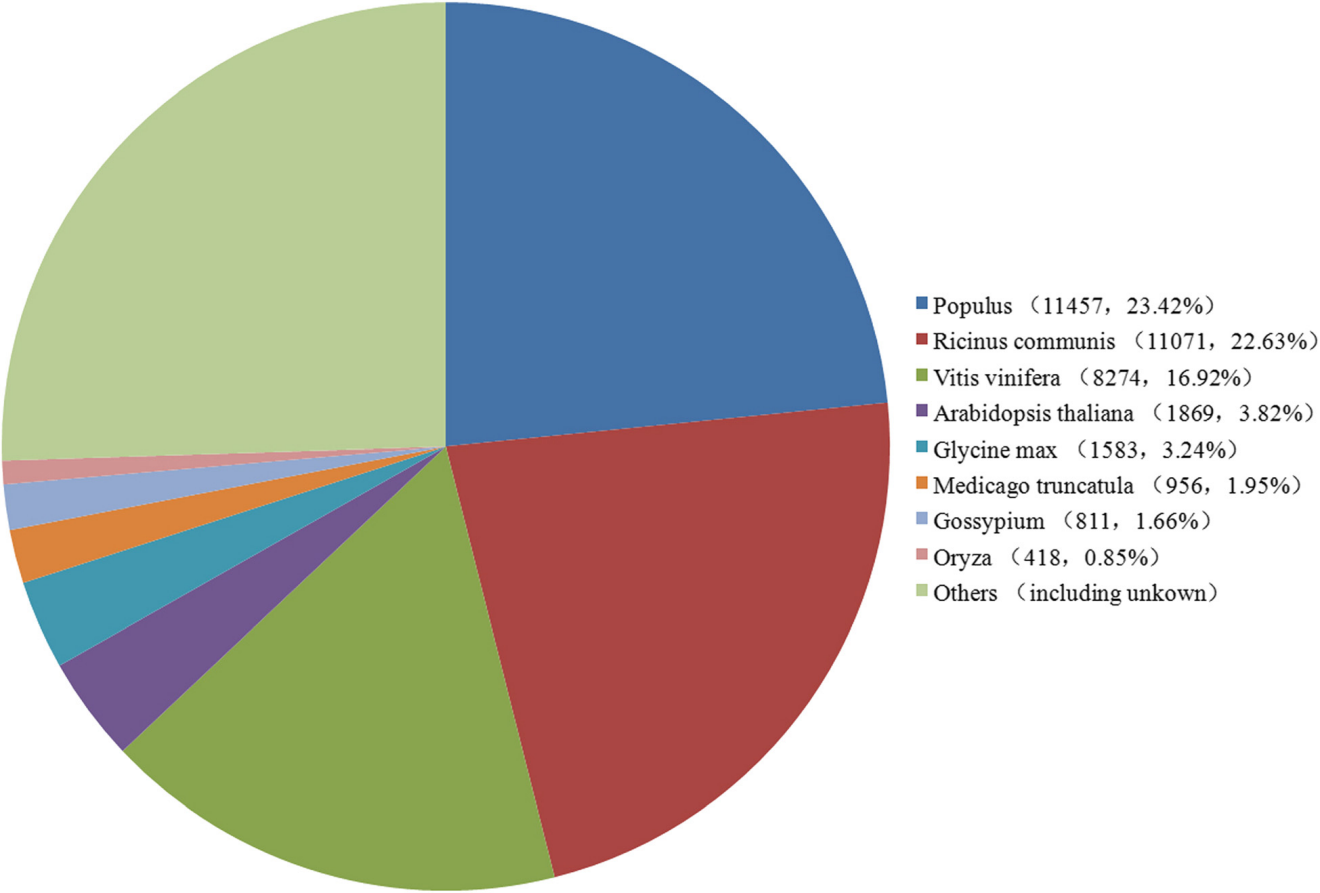
Percentage numbers of the eight most abundant annotated species

After functional annotation, the numbers of sequences from different species that matched jute unigenes were calculated from the annotation characteristics. As displayed in Fig. 2, the four most abundant species were *Populus* (11457, 23.42 %), *Ricinus communis* (11071, 22.63 %), *Vitis vinifera* (8274, 16.92 %), and *Arabidopsis thaliana* (1869, 3.82 %), representing around 63 % of all the species that were annotated, which is consistent to the previous transcriptome sequence of jute [11].

### GO annotation

**Fig. 3:**
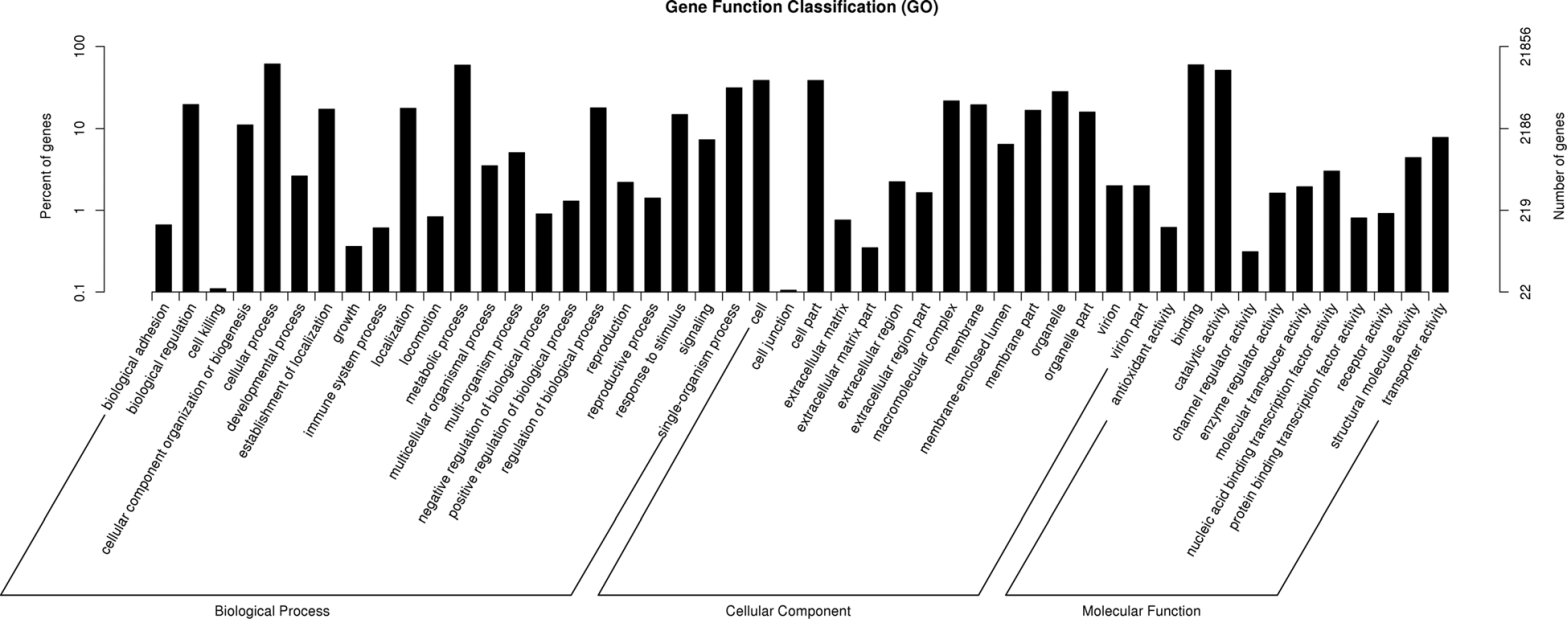
Gene ontology classifications of assembled unigenes in jute

Combined with the information from Nr annotation, GO annotation for the assembled unigenes was obtained using the Blast2GO software [17]. The classifications of GO functions were performed using the WEGO program [18]. Totally, 21,856 unigenes (44.68 %) were assigned to 56 classifications (Fig. 3). Among the 56 GO classifications, Cellular process (13,311) represented the largest group, followed by Binding (13,008), Metabolic process (12,914), Catalytic activity (11,166), Cell (8391), Cell part (8377) and Single-organism process (6797), whereas only a few unigenes were assigned to Nucleoid, Synapse part, Synapse, Receptor regulator activity, and Metallochaperone activity.

### KOG annotation

**Fig. 4:**
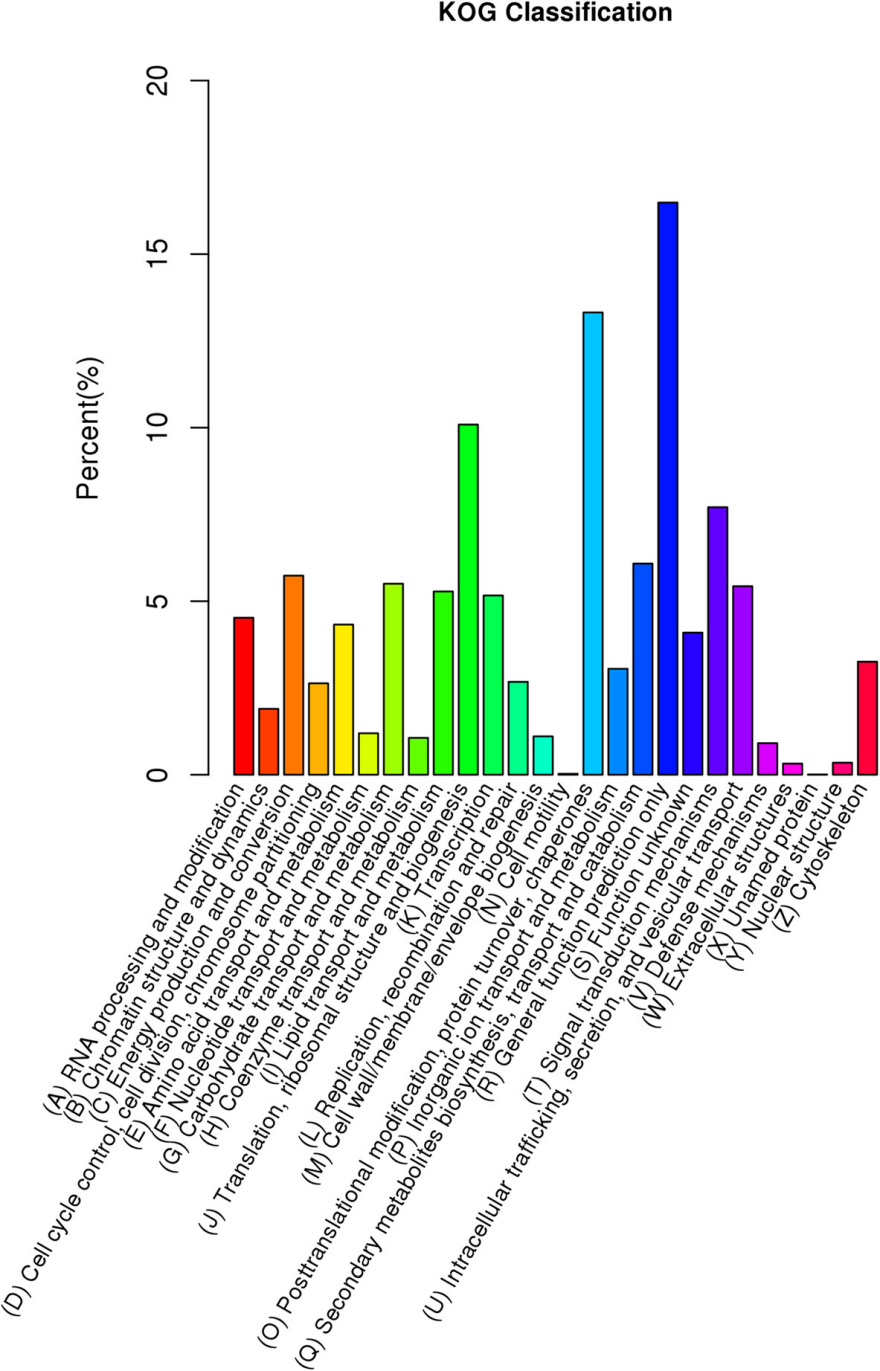
Histogram of clusters of eukaryotic ortholog groups (KOG) classification in jute. The horizontal axis is the name of clusters of KOG; and the vertical axis is the proportion of the group to the total number

To assess the validity and integrity of the transcriptome sequence, 25,739 unigenes annotated in Nr database were assigned to the KOG database to classify potential functions. In total, 11,190 genes were aligned to the 26 KOG classifications (Fig. 4). Among them, assignments to (R) General Functional Prediction made up the majority (1845, 16.49 %), followed by (O) Post-translational modification, protein turnover, chaperon (1491, 13.32 %), (J) Translation, ribosomal structure and biogenesis (1129, 10.09 %) and (T) Signal Transduction mechanisms (863, 7.71 %). But assignments to (N) Cell motility (3, 0.03 %), (W) Extracellular structures (36, 0.32 %) and (Y) Nuclear structure (39, 0.35 %) made up the minority.

### KEGG annotation

**Fig. 5:**
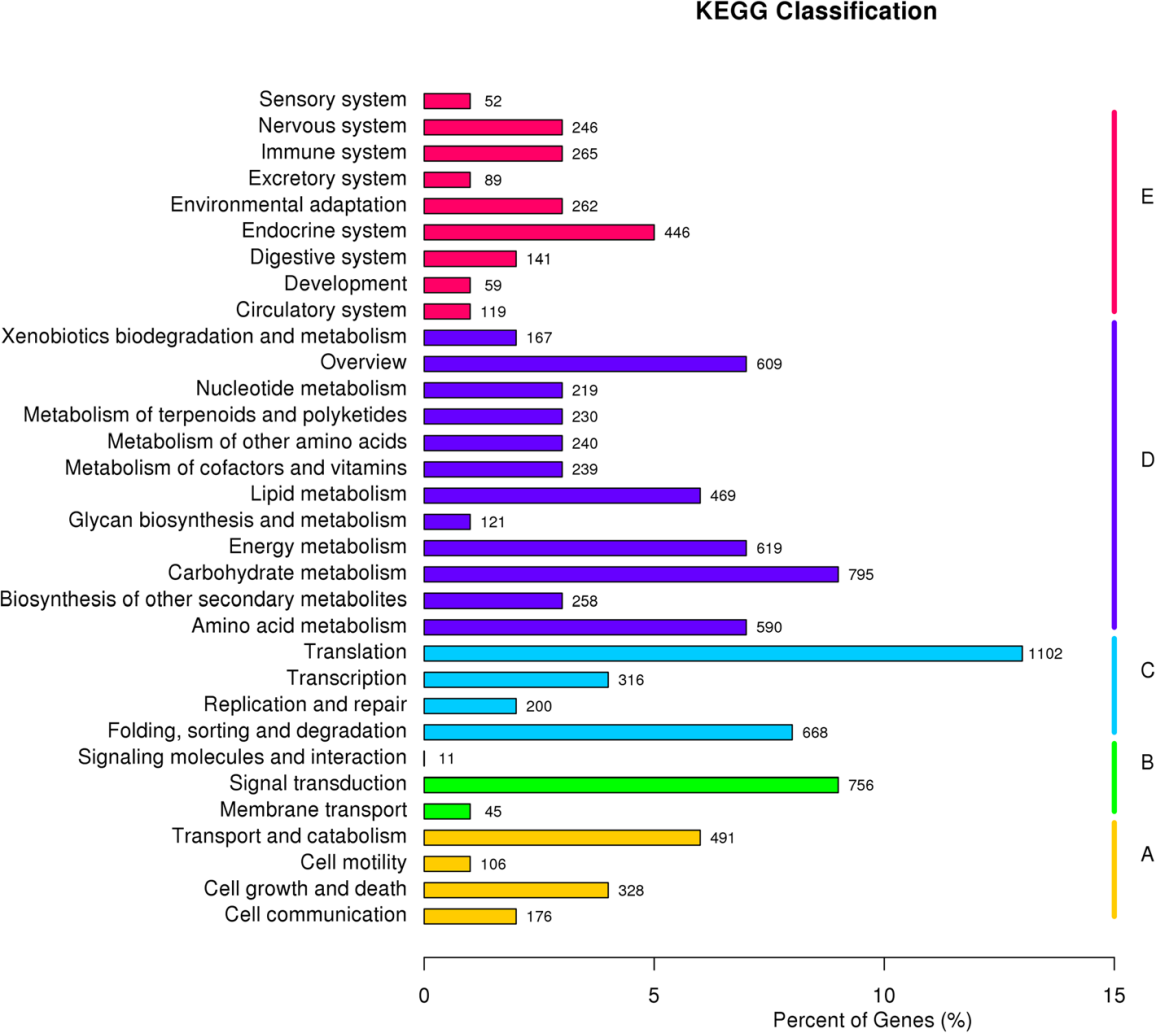
Histogram of clusters of kyoto encyclopedia of genes and genomes (KEGG) in jute. The horizontal axis is the proportion of the group to the total number; and the vertical axis is the name of clusters of KEGG. According to KEGG metabolic pathway, The genes could be divided into five branches: cellular processes (A), Environmental Information Processing (B), genetic information processing (C), metabolic (D), and organismal system (E)

To understand the interaction of genes and metabolic biological functions, 14,216 unigenes, which had significant matches in the Kyoto Encyclopedia of Genes and Genomes (KEGG) database using BLASTx with an E-value threshold of 10^−5^, were assigned to 268 pathways (Fig. 5). There are five KEGG categories based on pathway hierarchy 1 (Additional file 3) including Cellular Processes (A) (1538), Environmental Information Processing (B) (1368), Genetic Information Processing (C) (2560), Metabolism (D) (6214), and Organismal Systems (E) (2536) respectively. From the ratio of the five classifications, we can see that Metabolism (D) and Genetic Information Processing (C) have the greatest number of unigenes. Among the pathway subgroups based on pathway hierarchy 2 (Additional file 3), the pathway of Ribosome was the most unigenes (662), followed by Carbohydrate metabolism(328), Biosynthesis of amino acids(327), Protein processing in endoplasmic reticulum (288) (Additional file 3).

Because cellulose is one of the main components in jute fiber, starch and sucrose metabolic pathway (ko00500) which was related with cellulose biosynthesis should be of particular interest. There are 172 unigenes which were assigned to starch and sucrose metabolic pathway. Likewise, lignin, which is a complex polyphenolic polymer, is another main component of jute fiber. There are 67 and 143 unigenes which involved in pathways of phenylalanine, tyrosine and tryptophan biosynthesis (ko00400, i.e., shikimate-AAA), and monolignol biosynthesis (ko00940) respectively.

### CDS prediction

**Fig. 6:**
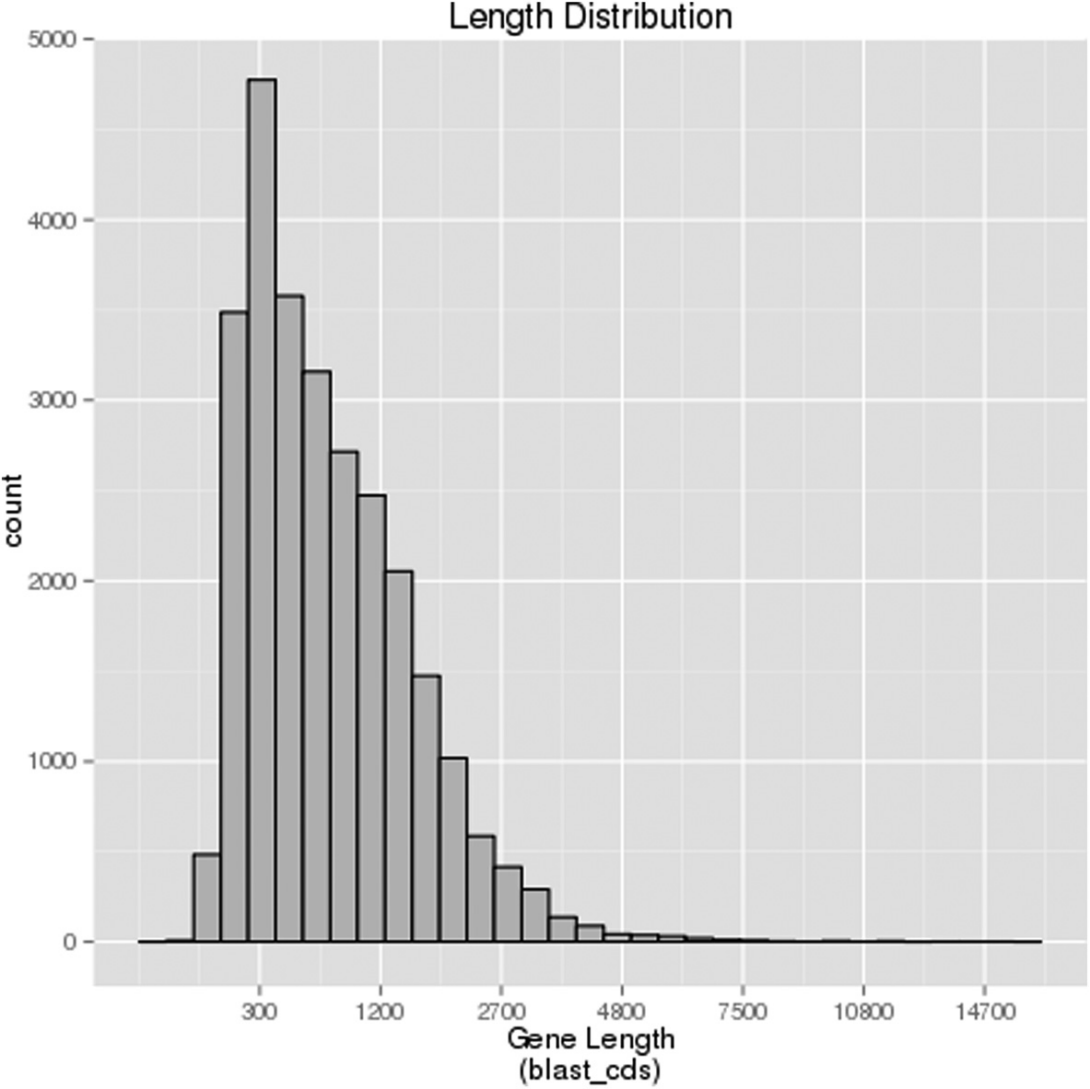
Length distribution of unigenes predicted protein coding sequence (CDS) using BLAST (Basic Local Alignment Search Tool) in jute

**Fig. 7:**
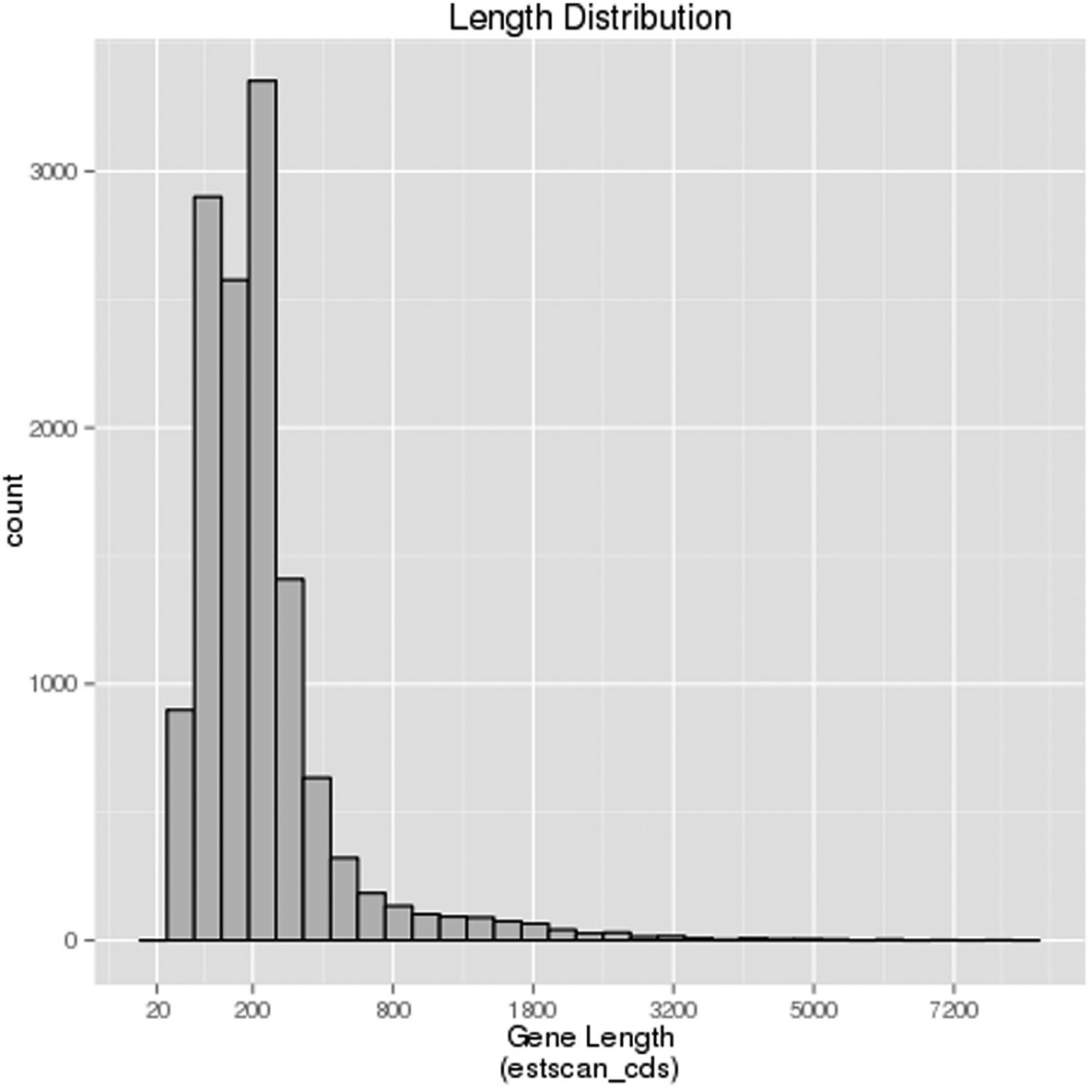
Length distribution of assembled unigenes predicted protein coding sequecnce (CDS) using EST Scan in jute

Coding sequecnce (CDS) of the de novo assembled unigenes were predicted first using BLASTX, then EST Scan. A total of 26,899 (54.99 %) unigenes were predicted via BLASTX with an E-value threshold of 10^−5^ in the non-redundant protein (Nr), the Swiss-Prot protein, and the KEGG database (Figs. 6 and 7). Among these, 20,757 unigenes were in the length of more than 300 bp (Fig. 6). Furthermore, 12,994 (25.8 %) unigenes were then predicted using EST Scan, which identified 2687 unigenes of more than 300 bp in length (Fig. 7). However, 9012 (18.44 %) of these unigenes had no CDS matches so far.

### SNP calling

GATK2 software was used to call SNP. As summarized in Table 3, a total of 12,518 SNPs putatively associated with genes were identified from assembled jute sequences (Additional file 4).

**Table 3:** Frequencies of SNPs from unigenes based on the transcriptome sequence in jute

Totle SNP	Non coding SNP	Coding SNP	Synonymous	Nonsynonymous
12,518	8863(70.80 %)	3655(29.20 %)	3632	23

Among the 12,518 SNPs, the SNP types of transition and transversion were identified at frequencies of 59.2 and 22.3 % respectively (Table 4). The most abundant transition SNPs were G/A and C/T. The most transversion SNPs were A/T and G/T. Of these identified SNPs, the noncoding and coding SNP types have frequencies of 70.80 % (8863) and 29.20 % (3655) respectively (Table 3), indicating that SNPs occur in noncoding regions more frequently than in coding regions. Since gene-associate SNPs could have effects on traits, the frequencies of synonymous (with no effect on protein sequence) and nonsynonymous SNPs (that affect protein sequence) were determined. Of the coding SNPs, the frequency of the synonymous SNP type was 99.37 %. The SNPs identified here will contribute to the development of a high-density SNP array.

**Table 4:** Frequencies of SNP types from unigenes based on the transcriptome sequence of jute

SNP type	SNP	Numbers
Transition		7415(59.2 %)
	C/T	3536
	G/A	3879
Transversion		5103(22.3 %)
	C/A	1190
	G/T	1214
	C/G	1026
	A/T	1673

### Identification of major bast related genes involved in cellulose biosynthesis

**Fig. 8:**
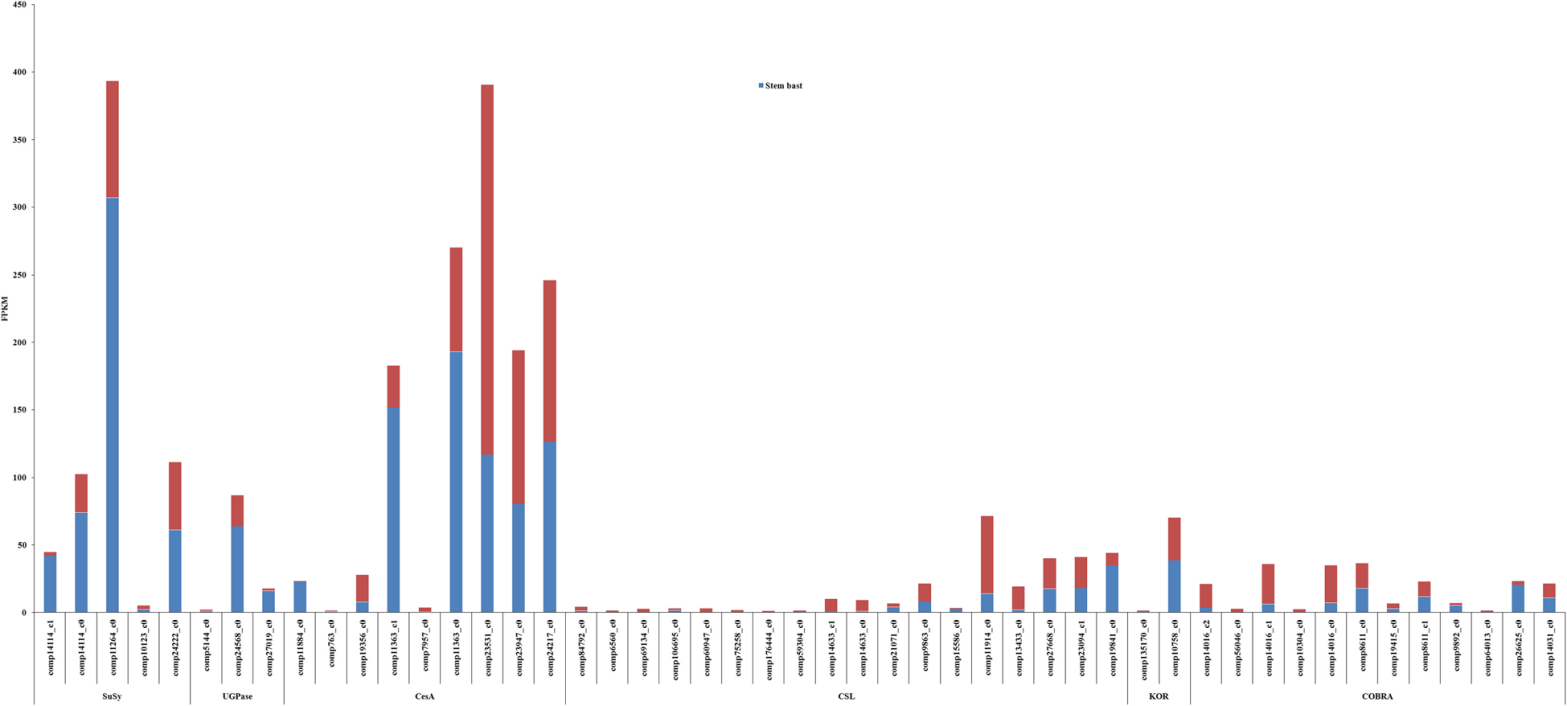
The expression pattern of major genes involved in cellulose biosynthesis in jute. The FPKM of X axis (blue plus red) represents gene expression of the mixed tissues of stem bast, stem stick, leaf and root; The FPKM of X axis (blue: Stem bast) represents gene expression of stem bast

The cellulose is one of principal components of the cell walls, and consists of glucose residue. According to natural fiber crops such as ramie [7, 8], flax [9], hemp [10], the candidate genes corresponding to fiber development, such as sucrose synthase (*SuSy*), UDP-glucose pyrophosphorylase (*UGPase*), cellulose synthase (*CesA*), cellulose synthase-like (*CSL*), *KOR* (*KORRIGAN*), and *COBRA*, were determined. Of 48,914 assembled unigenes, 27,962 unigenes were annotated in at least one public protein database. By searching the annotation of 27,962 unigenes with the expression of sucrose synthase, UDP-glucose pyrophosphorylase, cellulose synthase, korrigan, and cobra, 5 *Susy*, 3 *UGPase*, 9 *CesA*, 18 *CSL*, 2 *KOR*, and 12 *COBRA* unigenes were identified (Additional file 5). To identify the expression profile of the candidate genes associated with cellulose biosynthesis, the transcript abundance of all the candidate genes in the mixed tissue (leaf, root, stem bast and stem stick) and stem bast were investigated by using FPKM analysis (Fig. 8). RT-qPCR experiments of randomly selected genes confirmed the expression level of genes of interest using FPKM (Additional files 6, 7 and 8).

#### SuSy

Sucrose synthase, which is an integral component of the cellulose synthesis machinery, participates in starch and sucrose metabolic pathway. For *Susy*, five unigenes were identified, with their sequence lengths ranging from 1237 to 3152 bp. Among them, the unigene of comp11264_c0 displayed the highest expression level while comp10123_c0 showed the lowest expression level both in the mixed tissues and stem bast. All of them were assigned to starch and sucrose metabolic pathway, which are worthy of doing in-depth research.

#### UGPase

*UGPase* is a precursor of catalytic cellulose-uridine diphosphate glucose (UDP-glucose, UDPG) synthesis. For *UGPase*, 3 unigenes, i.e., comp5144_c0, comp24568_c0, and comp27019_c0, were identified. The sequence lengths of comp5144_c0, comp24568_c0, and comp27019_c0 are 282, 1885, and 2356 bp respectively. Among of them, comp24568_c0 showed the higher expression level than the other two unigenes both in the mixed tissues and stem bast. Like *SuSy*, all of them were assigned to starch and sucrose metabolic pathway.

#### CesA and CSL

The catalytic subunits of *CesAs* are central catalysts involving in the generation of plant cell wall cellulose. For *CesAs*, nine unigenes, the sequence lengths of whose varied from 311 to 3873 bp, were identified. Of them, the unigene of comp11363_c0 displayed the highest expression level in the stem bast, followed by comp11363_c1, comp24217_c0, comp23531_c0. For *CSLs*, 18 unigenes, were identified. The sequence lengths ranged from 229 to 4418 bp. Compared with the unigenes of *CesAs*, most of the unigenes of *CSLs* showed the low expression level in the stem bast, showing that *CesAs* plays more important role in the fiber formation than *CSLs* at the vegetable growth stage.

#### KOR (KORRIGAN)

*KOR* severed cellulose chains to terminate microfibrils. For *KORs*, two unigenes of comp135170_c0 and comp10758_c0 were identified. The sequence lengths of comp135170_c0 and comp10758_c0 are 293 and 1960 bp respectively. The unigene of comp10758_c0 showed the higher expression level than comp135170_c0 both in the mixed tissue and stem bast.

#### COBRA

***COBRAs*** are involved in the orientation of deposition of cellulose microfibrils. For ***COBRAs***, 12 unigenes, the sequence lengths of whose varied from 465 to 2624 bp, were identified. Most of them displayed the low expression level in the stem bast, comparing with the other gene families which are involved in cellulose synthesis.

Taken together, the expression level of most of the cellulose biosynthesis genes were relatively higher in stem bast than the other tissues. Expression quantities of *SuSy, UGPase,* and *CesA* were higher than that of other gene families, indicating that *SuSy, UGPase,* and *CesA* play important role in the fiber formation at a vigorous vegetable growth stage. The abundant expression of the unigenes of comp11264_c0 (*SuSy*), comp24568_c0 (*UGPase*), comp11363_c0 (*CesA*), comp11363_c1 (*CesA*), comp24217_c0 (*CesA*), comp23531_c0 (*CesA*) suggested that they have a principal role in the corresponding gene family.

## Discussion

### De novo assembly of 48,914 unigenes based on transcriptome sequence of jute

Recently, to accelerate the research of functional genomics and mine the genes involved in important agricultural traits, transcriptomic sequences of some minor crops have been characterized with NGS [4–11]. But, for jute, only 858 expressed sequence tags (EST) were deposited in GenBank database so far (as of June, 2015). The lack of gene sequences and functional annotation seriously hindered to dissect genetic basis of jute fiber development, which leads to an obstacle to jute improvement. In this study, the transcriptomic sequence of jute was characterized and 48,914 unigenes were discovered. Although Chakraborty et al. [11] reported a comparative bast transcriptomes from a *deficient lignified phloem fibre* mutant and its wild-type jute (*Corchorus capsularis*) using Illumina paired-end sequencing, the transcriptomic sequence in the present study is obviously different, because the RNA of both studies isolated different tissues. The RNA of this study isolated from different tissues of leaves, roots, stem bast, and stem stick at a vigorous vegetative stage; while the RNA of Chakraborty et al. [8] extracted from the bast fiber tissues excluding the inner core tissues. Thus, the objectives of both studies are also different. The aim of the present study was to provide a whole transcriptomic characterization at a vegetable growth stage using an elite variety; while the aim of Chakraborty et al. [8] was to explain the mutant of *deficient lignified phloem fibre* in terms of lignin and secondary cell wall.

Undoubtedly, this study is another good example to characterize the jue transcriptome, which achieved a great leap in the discovery of jute genes. The average length of 48,914 unigenes was 903 bp, which was longer than that of some other crops’ transcriptome sequences [19–22]. Moreover, 27,962 (57.16 %) unigenes were annotated in at least one database of Nr, Nt, Pfam, KOG/COG and Swiss-Prot. After annotation, it was found that *Populus*, *Ricinus communis*, and *Vitis vinifera* were the top three species, which was very similar to previous results for jute transcriptome sequencing [11]. In addition, 14,216 unigenes were assigned to 268 pathways based on KEGG database. A total of 172 unigenes were assigned to starch and sucrose metabolic pathway. Likewise, there are 67 and 143 unigenes involved in pathways of phenylalanine, tyrosine and tryptophan biosynthesis (ko00400, i.e., shikimate-AAA), and monolignol biosynthesis (ko00940) respectively. Therefore, we are convinced that this transcriptome sequence is a sufficient supplement for further genetic dissection important agricultural traits, especially bast fiber development mechanism in jute.

### SNP marker identification

The markers of SNP, which could be applicable in genetic and breeding studies in jute, are still limited so far. Although some SNP markers derived from jute have been reported [13], the lack of SNP markers, in a certain sense, has resulted in a large gap in the studies of marker-assisted breeding and genetic map construction. In this study, 12,518 SNPs were identified based on the transcription sequence, which greatly enriched the number of SNP in jute. As far as a plant, 12,518 SNPs is still not sufficient. The reason might be SNP detected on the basis of one transcriptome sequence rather than whole genome sequence. It also reflected that this jute transcriptome assembly will be further improved when more sequence data become available.

Among the 12,518 SNPs, the transition and transversion SNP types were identified at frequencies of 59.2 % and 22.3 % respectively, which revealed that transition SNPs types were more predominant (2:1) than transversions, similar to most of previous studies [13, 23]. Furthermore, the patterns of SNP types showed that G/A and C/T are the most common types. The high proportion of C/T transitions might be due to deamination of 5-methylcytosine reactions, which occurs frequently at CpG dinucleotides [24]. The frequencies of the noncoding and coding SNP types showed that SNPs occur in non-coding regions (70.80 %, 8863) more frequently than in coding regions (29.20 %, 3655), which is in accordance with most of previous studies on SNP development [13].

### Identification of genes involved in cellulose biosynthesis

Cellulose is one of the main components of bast fiber and regarded as one of the main objectives for improvement in natural fiber crops. A typical jute fiber has 13.5 % lignin, 15.9 % hemicellulose, and 61 % cellulose [1]. The relatively low cellulose content distinguishes jute fiber from other natural fiber of flax, hemp and ramie. For example, a typical ramie fiber has 1.5 % lignin, 14 % hemicellulose, and 70 % cellulose. For genetic improvement of lignocellulosic bast fibers, it is a good way by raising the cellulose. To date, it is widely accepted that there are several gene families which are required for cellulose synthesis: *SuSy*, *UGPase*, *CesA*, *CSL*, *KOR*, and *CORBA* [4, 7–10]. However, only each of *UGPase* and *CesA* genes in jute has been currently identified respectively using homologous cloning and RACE technology [25, 26]. In this study, 5 *Susy*, 3 *UGPase*, 9 *CesA*, 18 *CSL*, 2 *KOR* , and 12 *COBRA* unigenes were identified based on the transcriptome sequence. With the survey of the KEGG database, 5 *Susy* and 3 *UGPase* were found in the starch and sucrose metabolic pathway. Furthermore, the expression pattern analysis of these genes involving in cellulose biosynthesis showed that the expression level of most of the cellulose biosynthesis genes in stem bast were relatively higher than that in the other tissues. It indicated that these major cellulose biosynthesis genes play important role in the fiber formation at the vigorous growth stage. Compared with the expression pattern of the several gene families which are required for cellulose synthesis, it is interestingly found that the expression level of *Susy*, *UGPase,* and *CesAs* in stem bast were higher than that of *CSL, KOR,* and *COBRA,* giving a hint that *Susy*, *UGPase,* and *CesAs* might play more important role in the fiber formation at a vigorous growth stage than *CSL, KOR,* and *COBRA*. Of these genes involving in cellulose biosynthesis, the unigenes of comp11264_c0 (*SuSy*), comp24568_c0 (*UGPase*), comp11363_c0 (*CesA*), comp11363_c1 (*CesA*), comp24217_c0 (*CesA*), comp23531_c0 (*CesA*) highly expressed in the stem bast. It is possible that these genes with high expression level in the stem bast are responsible for the cellulose biosynthesis. Therefore, identification of genes involved in cellulose biosynthesis for jute bast fiber will facilitate the dissecting the genetic basis and molecular mechanisms of fiber development.

## Conclusion

In this study, we characterized the transcriptome of an elite jute variety at the vegetable growth stage and discovered 48,914 unigenes, which generates a broad survey of genes associated with vegetative growth and development. And we developed 12,518 putative gene-associate SNPs, which will contribute to the development of high-density genetic maps and SNP arrays. Furthermore, we analyzed the expression patterns of major genes involved in cellulose biosynthesis for bast fiber, including 5 *Susy*, 3 *UGPase*, 9 *CesA*, 18 *CSL*, 2 *KOR*, and 12 *COBRA* unigenes, which will provide a valuable knowledge to study cellulose biosynthesis. The trancriptome sequence here is a valuable genomics resource for jute research.

## Methods

### Plant material and RNA isolation

An elite white jute cultivar ‘Huangma 179’ was planted at the experimental farm of Fujian Agricultural and Forestry University, Fuzhou, China on May 1st, 2013. Various tissues, including leaves, roots, stem bast, and stem stick, were collected from three plants on June 15th, 2013, which is a vigorous vegetative stage for fiber development in jute according to Xiong [1]. Three independent biological replicates of each tissue sample were collected and immediately frozen in liquid nitrogen. Total RNA was isolated from each tissue sample respectively using TRIzol reagent (Life Technologies, CA, US) according to the manufacturer’s instructions as described by Zhang et al. [26]. The purity and content of each RNA was measured using Qubit RNA Assay Kit in Qubit 2.0 Flurometer (Life Technologies, CA, USA) and confirmed by 1 % agarose gels.

### Transcriptome sample preparation and sequence

There are two sample sequencing data in the current manuscript. One paired-end (PE) cDNA library was generated from pooled equal quantities of total RNA from stem bast, stem stick, leaves, and roots. Another paired-end (PE) cDNA library was generated from stem bast. Illumina sequencing was conducted at Beijing Novogene Biological Information Technology Co., Ltd., Beijing, China (http:// www.novogene.cn/) by using Illumina TruSeq™ RNA Sample Preparation Kit (Illumia, San Diego, USA) following manufacturer’s recommendations [27]. Briefly, mRNA was purified from total RNA using poly-T oligo-attached magnetic beads. Fragmentation was carried out using divalent cations under elevated temperature in Illumina proprietary fragmentation buffer. First strand cDNA was synthesized using random oligonucleotides and SuperScript II. Second strand cDNA synthesis was subsequently performed using DNA Polymerase I and RNase H. Remaining overhangs were converted into blunt ends via exonuclease/polymerase activities and enzymes were removed. After adenylation of 3’ ends of DNA fragments, Illumina PE adapter oligonucleotides were ligated to prepare for hybridization. In order to select cDNA fragments of preferentially 200 bp in length the library fragments were purified with AMPure XP system (Beckman Coulter, Beverly, USA). DNA fragments with ligated adaptor molecules on both ends were selectively enriched using Illumina PCR (polymerase chain reaction) Primer Cocktail in a 10 cycle PCR reaction. Products were purified (AMPure XP system) and quantified using the Agilent high sensitivity DNA assay on the Agilent Bioanalyzer 2100 system.

Clustering of the index-coded samples was performed on a cBot Cluster Generation System using TruSeq PE Cluster Kit v3-cBot-HS (Illumia) according to the manufacturer’s instructions. After cluster generation, the library preparations were sequenced on an Illumina Hiseq 2000 platform and 90 bp paired-end reads were generated.

### Transcriptome assembly

Raw sequence data reads in fasta format were firstly processed through in-house perl scripts [14]. In this step, clean data(clean reads) were obtained by removing reads containing adapter, ploy-N and low-quality reads from raw read data. At the same time, Q20 and GC-content of the clean data were calculated. All the downstream analyses were based on clean data with high quality.

A flow chart of transcriptome assembly as described by Grabherret al. [28] was used in the present analyses. A Perl pipeline as described by Haas et al. [14] was used for analyzing sequence data. As suggested by Haas et al. [14], if multiple sequencing runs are conducted for a single experiment, these reads may be concatenated into two files in the case of paired-end sequencing. The left files (read1 files) from all samples were pooled into a single large left.fq file, and right files (read2 files) into a single large right.fq file. Transcriptome assembly was accomplished based on the left.fq and right.fq using Trinity [14] with min_kmer_cov set to two by default and all other parameters set default. For one paired-end(PE) cDNA library (left.fq and right.fq), which was generated from pooled equal quantities of total RNA from stem bast, stem stick, leaves, and roots, the assembled sequence data have been deposited at the NCBI Sequence Read Archive (SRA, http://www.ncbi.nlm.nih.gov/Traces/sra) under the accession number SRP060467 vide BioSamples SRS980707. For a sample sequencing data from stem bast (CK_JSB), the SRA files bearing with the accession number SRP060467 vide BioSamples SRS1047357 are deposited in NCBI.

### Gene functional annotation

Gene function was annotated using BLASTx (E value < 10^−5^) queries in the following databases: nr (non-redundant protein sequences, http://www.ncbi.nlm.nih.gov), Pfam (annotated Protein family), and Swiss-Prot (the manually annotated part of the UniProt protein sequence database, http://www.expasy.ch/sprot). Using annotations from the nr database, the Blast2GO program was used to obtain GO annotations (Gene Ontology, http://www.geneontology.org) according to molecular function, biological process, and cellular component ontologies [17]. GO functional classifications were performed using the WEGO program [18]. Unigene sequences were aligned to the KOG/COG (Clusters of Orthologous Groups of proteins, http://www.ncbi.nlm.nih.gov/COG) to predict and classify possible functions. Pathway assignments were carried out according to the Kyoto Encyclopedia of Genes and Genomes pathway database (KEGG, http://www.genome.jp/kegg).

### SNP calling

Picard v1.41 and SAM v0.1.18 were used to sort, remove duplicated reads, and merge the binary alignment map(BAM) results for each sample. GATK2 software was used to perform SNP calling [29, 30]. Raw VCF files were filtered with the GATK standard filter method and other parameters (cluster Window Size: 10; MQ0 ≥ 4 and (MQ0/(1.0*DP)) > 0.1; QUAL < 10; QUAL < 30.0 or QD < 5.0 or HRun > 5). SNPs with quality scores of less than 30 or fewer than 5 bp from end of sequence read were excluded from further analysis. Because jute is diploid and the inbred line ‘Huangma 179’ has been selfed for 12 generations, we can be confident that these results are not artifactual.

### FPKM and RT-qPCR of genes involved in cellulose biosynthesis

The RNA extracted from a mixed tissue (leaf, root, stem bast and stem stick) and stem bast was used for RT-qPCR. cDNA was obtained using a reverse transcription kit (TaKaRa PrimeScript™, China). Transcript abundances were calculated using Cufflinks version 0.9.3 [31] and the output normalized expression values in FPKM (Fragments Per Kilobase of exon per Million fragments mapped) analogous to single-read FPKM [32] were used for further comparative analysis. To confirm the expression level using FPKM, six genes were randomly chosen as cases for PCR validation (Additional files 7 and 8). RT-qPCR was performed using an ABI 7500 fluorescence quantitative PCR instrument. Each reaction contained cDNA (1 μL), gene-specific primers (0.5 μL, 10 μmol L^−1^) and 2× SYBR Green Master Mix (10 μL) in a final volume of 20 μL. The jute 18S rRNA gene was served as the endogenous control [25]. The primer sequences of genes involved in cellulose biosynthesis were showed in Additional file 6. Real-time quantitative PCR analysis was as follows: 95 °C for 10 min, followed by 40 cycles of 95 °C for 15 s, 55 °C for 20s and 72 °C for 30s. All experiments were used in triplicate for each sample and relative gene expression levels were calculated using the 2^−ΔΔCT^ method. All data was analyzed using Excel.

## Additional files

Additional file 1:
**Length distribution and characterization of assembled transcripts in jute.** (PNG 54 kb) Additional file 2:
**Length distribution and characterization of assembled unigenes in jute.** (PNG 51 kb) Additional file 3:
**Pathway assignment based on Kyoto Encyclopedia of Genes and Genomes (KEGG) pathway database.** (XLS 241 kb) Additional file 4:
**Calling 12,518 SNPs based on raw VCF files from the transcription sequence of jute with the GATK standard filter method.** (XLS 2092 kb) Additional file 5:
**Major genes involved in cellulose biosynthesis based on the transcriptome sequence of jute.** (XLS 32 kb) Additional file 6:
**Primer sequences of randomly selected genes involving in cellulose biosynthesis and 18S RNA genes used for RT-qPCR.** (XLS 19 kb) Additional file 7:
**Comparison of expressional profile of randomly selected genes involving in cellulose biosynthesis using FPKM and RT-qPCR.** (JPG 141 kb) Additional file 8:
**A linear correlation diagram of expressional profile of randomly selected genes involving in cellulose biosynthesis between FPKM and RT-qPCR.** (JPG 121 kb)

## Abbreviations

BLAST: Basic Local Alignment Search Tool
CDS: coding sequecnce
CesA: cellulose synthase A
EST: expressed sequence tag
GO: gene ontology
KEGG: Kyoto Encyclopedia of Genes and Genomes Pathway database
KOG: euKaryotic Ortholog Groups
NGS: next generation sequencing
Nr database: NCBI non-redundant protein database
RT-qPCR: Real-time quantitative polymerase chain reaction
