# Supplementary figures and images for: De novo transcriptome sequence and identification of major bast-related genes involved in cellulose biosynthesis in jute (Corchorus capsularis L.)
Source: BMC Genomics. 2015 Dec 15;16:1062. doi: 10.1186/s12864-015-2256-z (PMC4678609; doi:10.1186/s12864-015-2256-z)

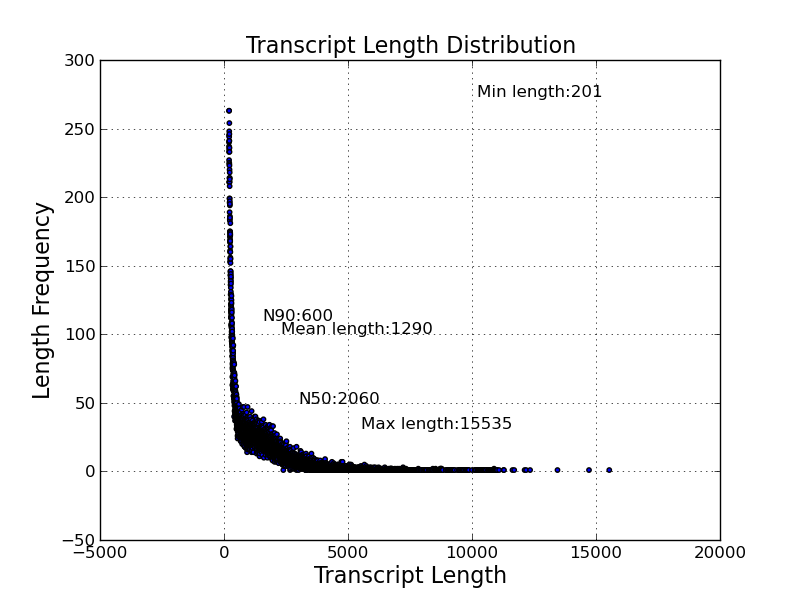

Supplement: Additional file 1: — Length distribution and characterization of assembled transcripts in jute. (PNG 54 kb) [file 12864_2015_2256_MOESM1_ESM.png]

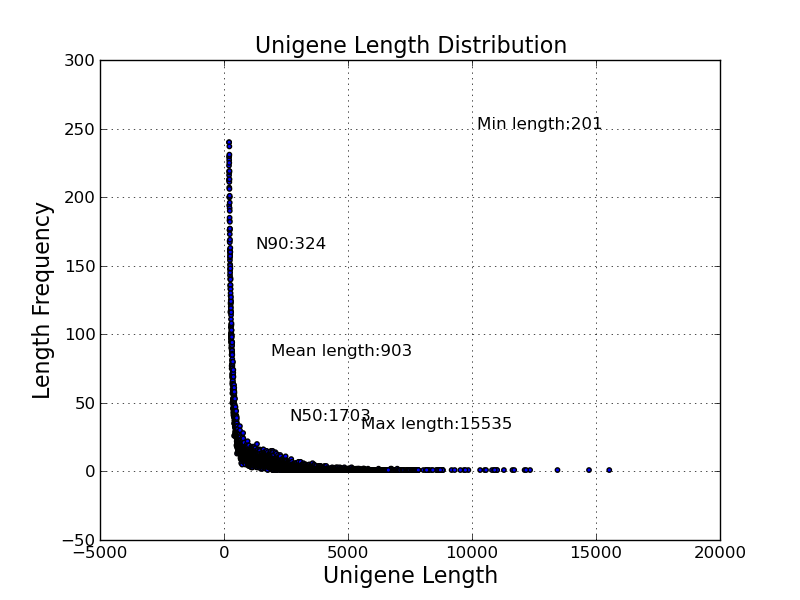

Supplement: Additional file 2: — Length distribution and characterization of assembled unigenes in jute. (PNG 51 kb) [file 12864_2015_2256_MOESM2_ESM.png]

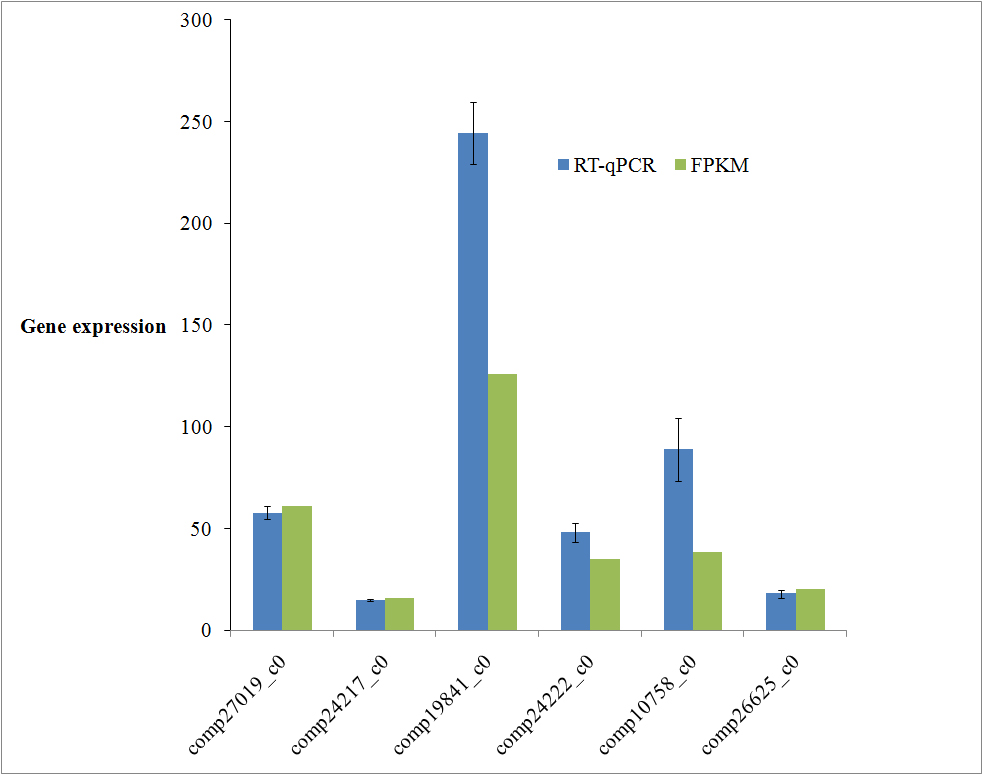

Supplement: Additional file 7: — Comparison of expressional profile of randomly selected genes involving in cellulose biosynthesis using FPKM and RT-qPCR. (JPG 141 kb) [file 12864_2015_2256_MOESM7_ESM.jpg]

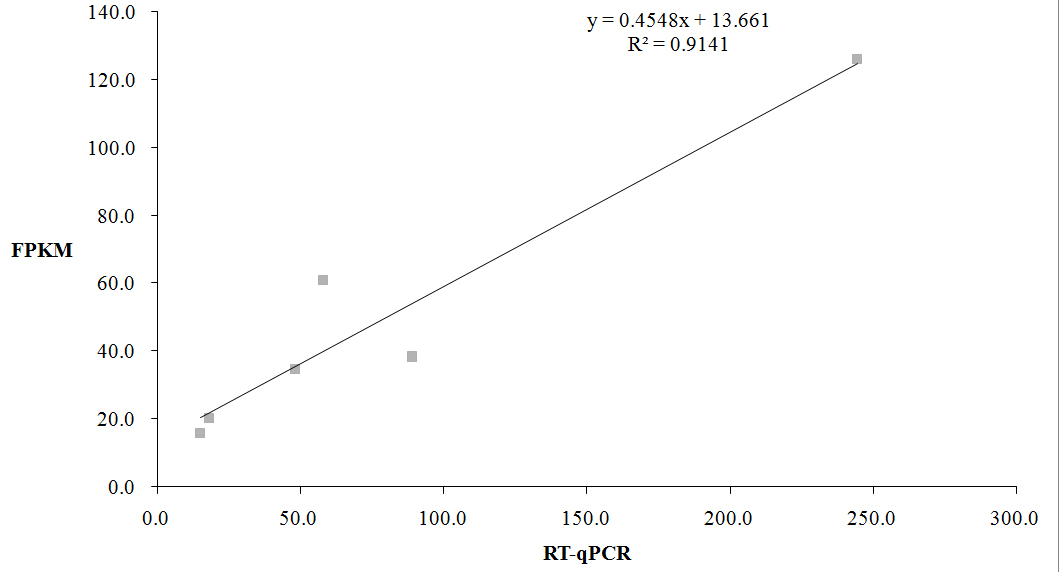

Supplement: Additional file 8: — A linear correlation diagram of expressional profile of randomly selected genes involving in cellulose biosynthesis between FPKM and RT-qPCR. (JPG 121 kb) [file 12864_2015_2256_MOESM8_ESM.jpg]
